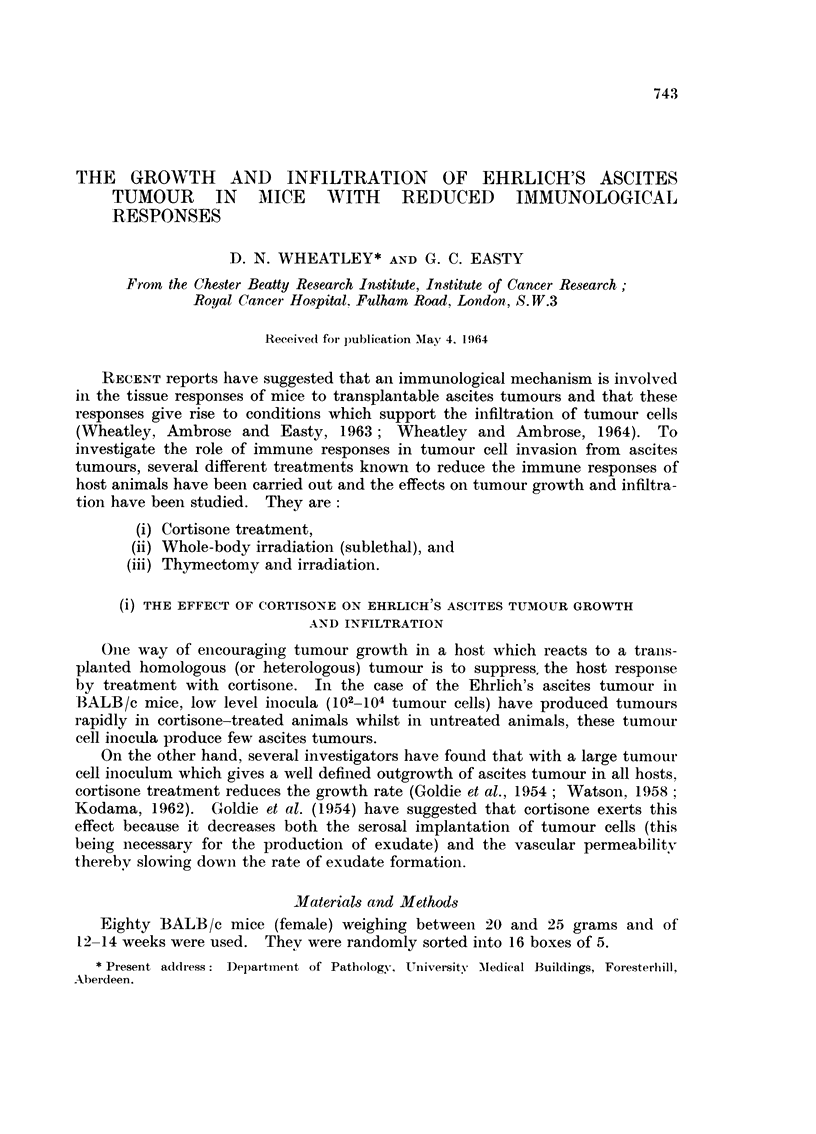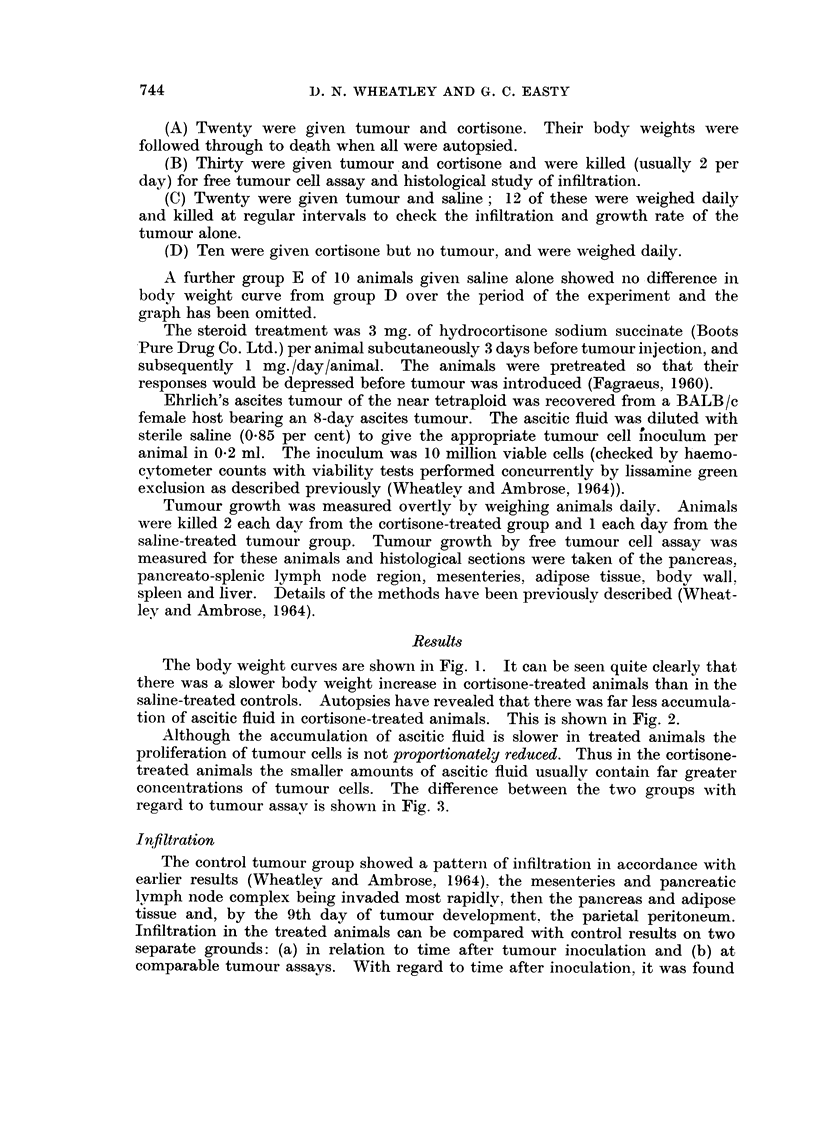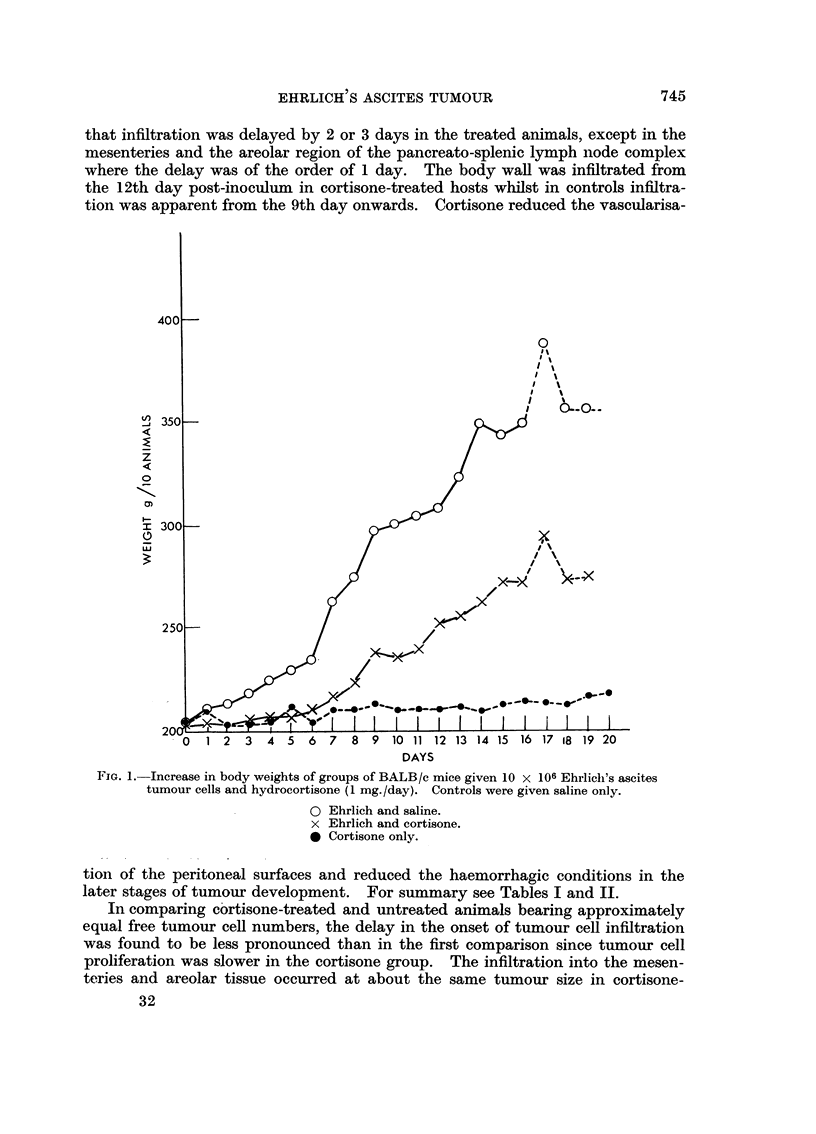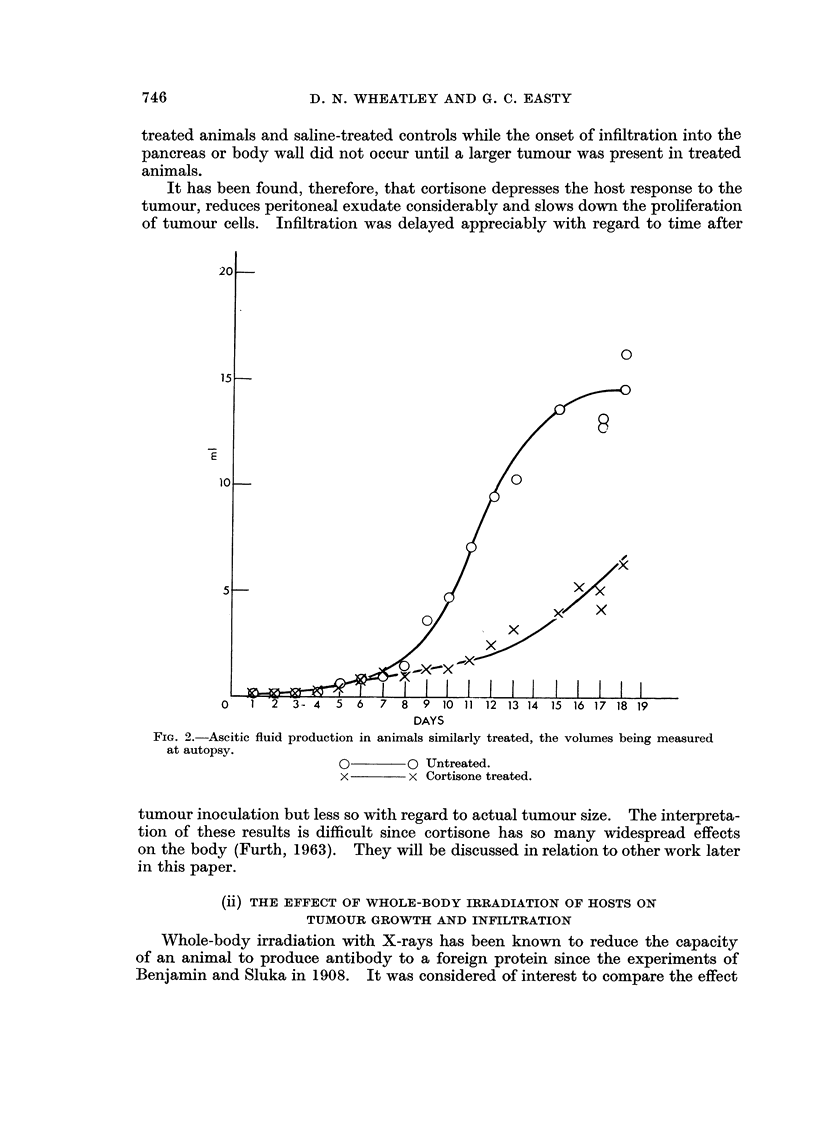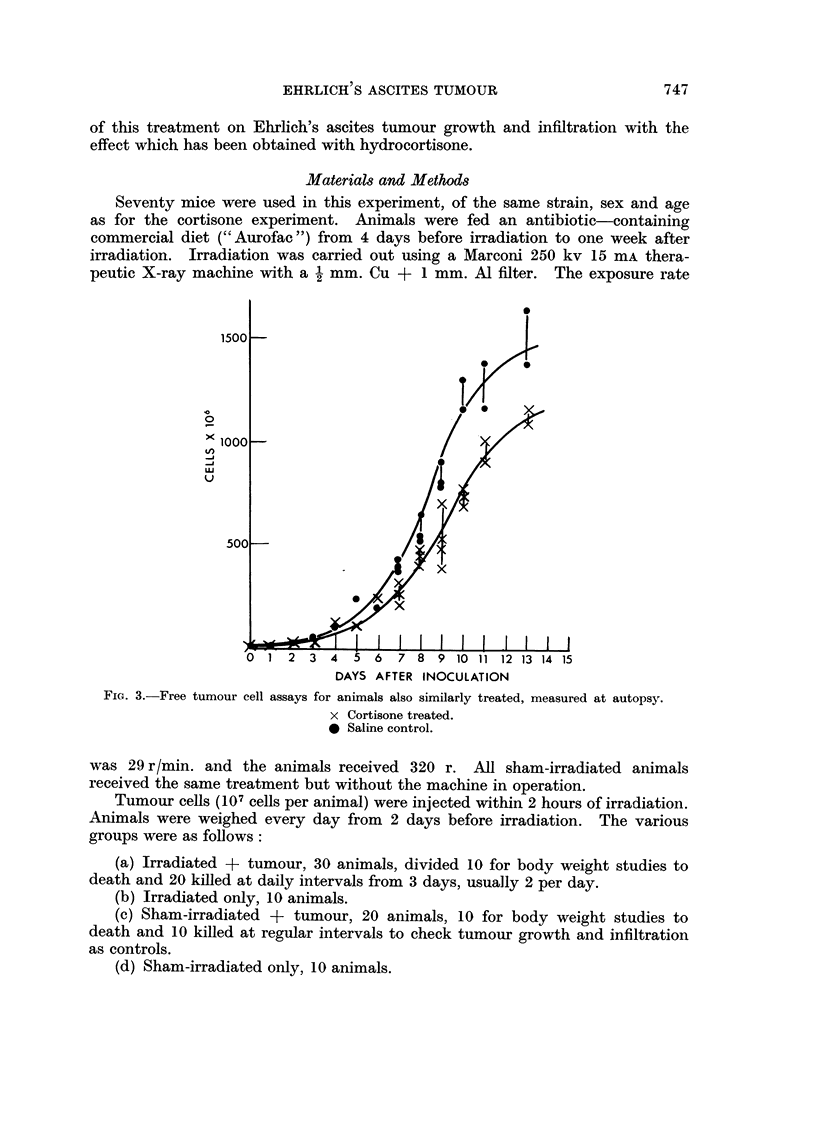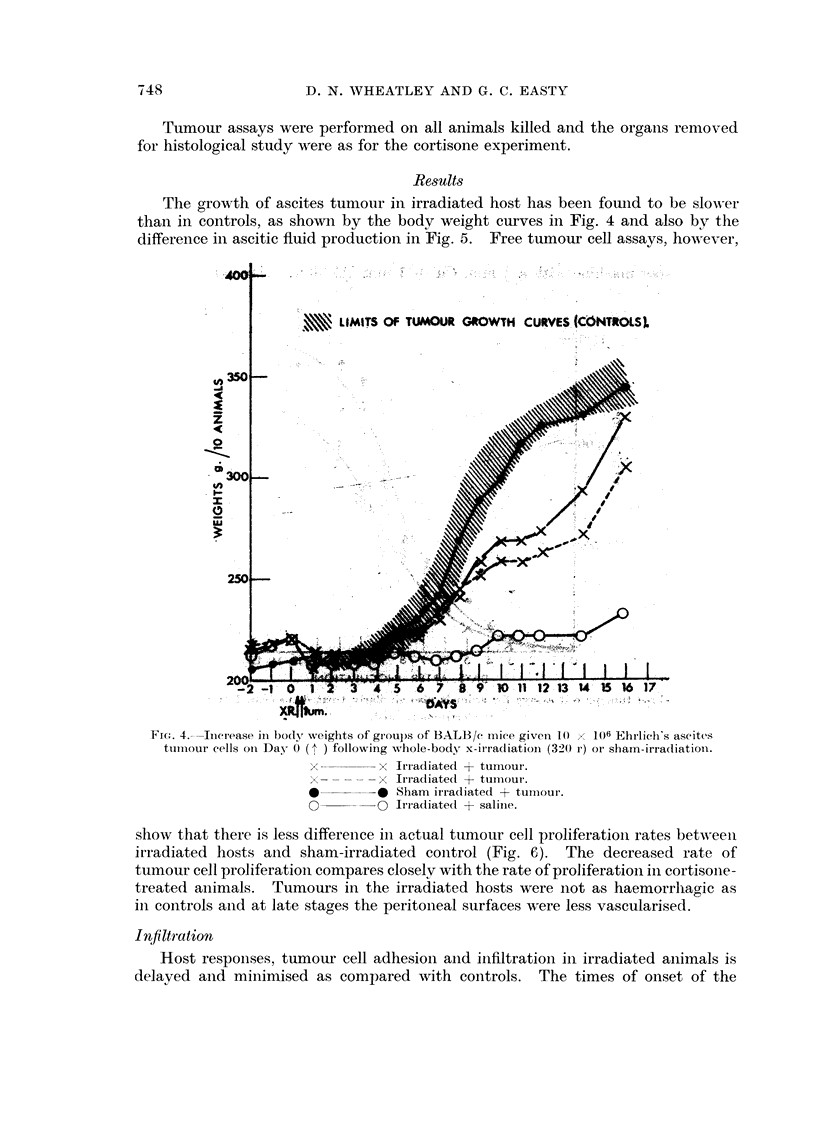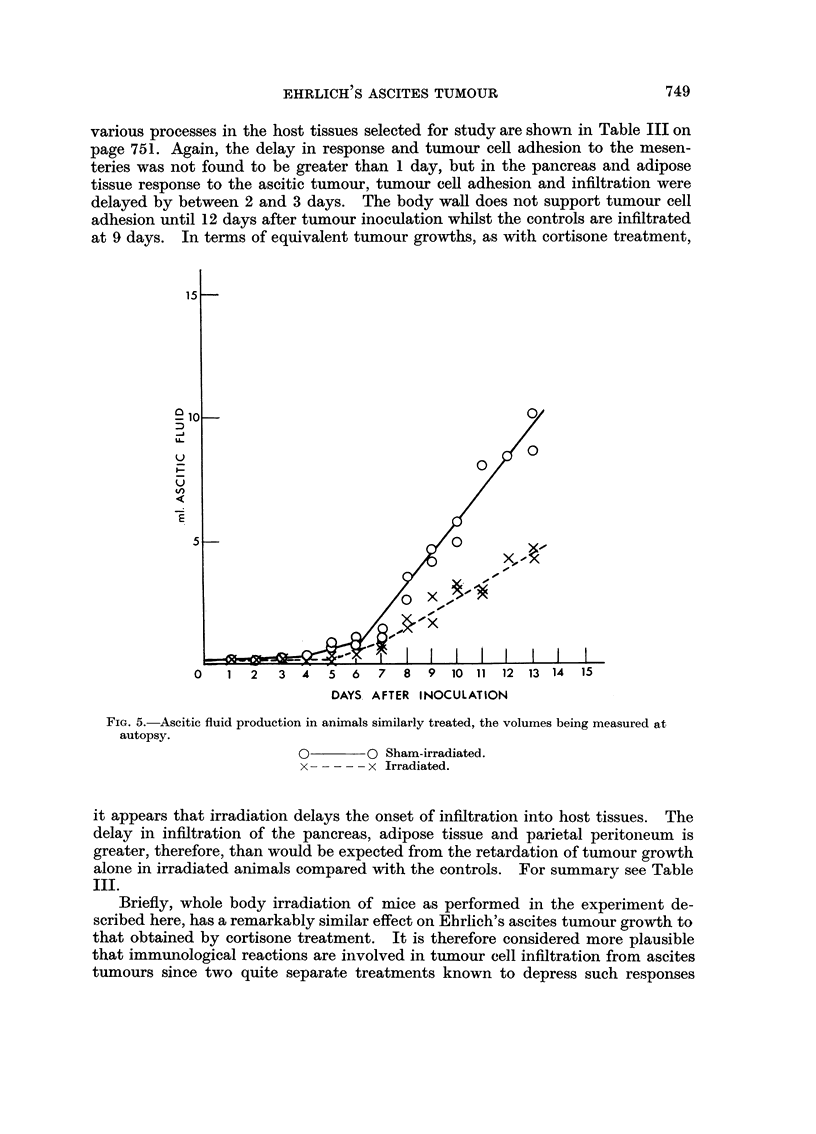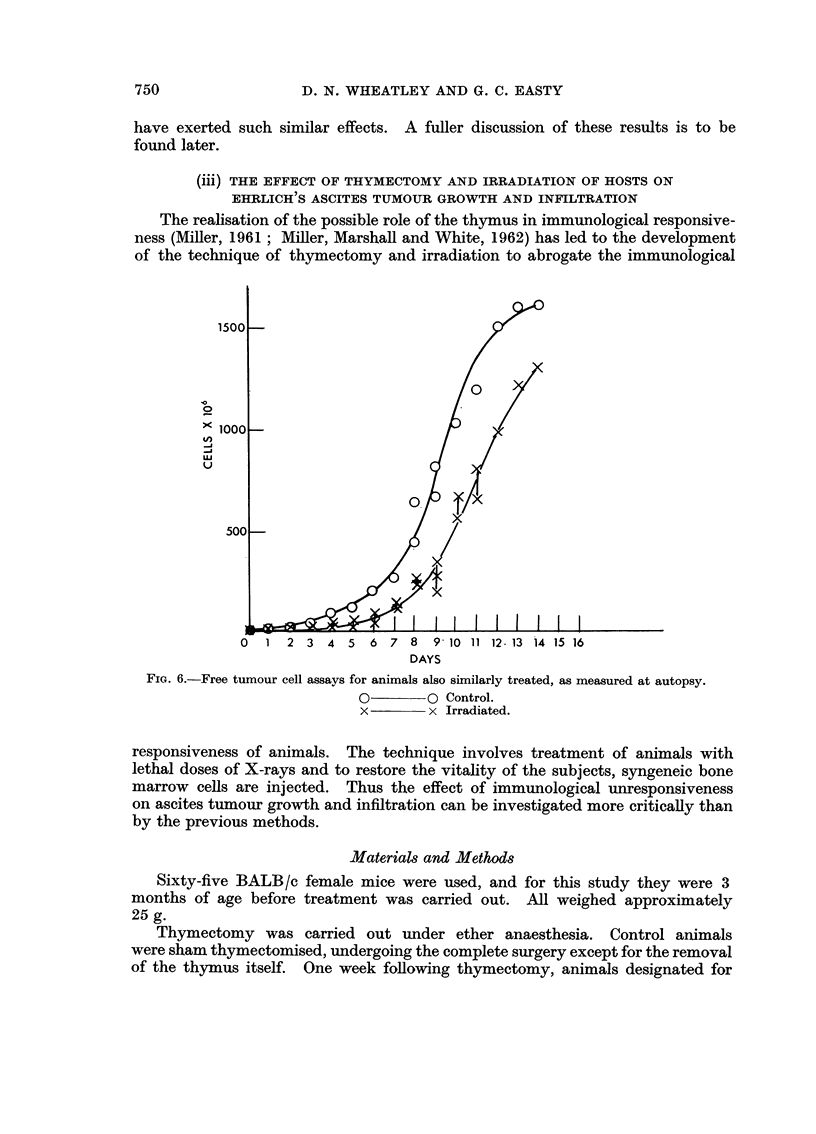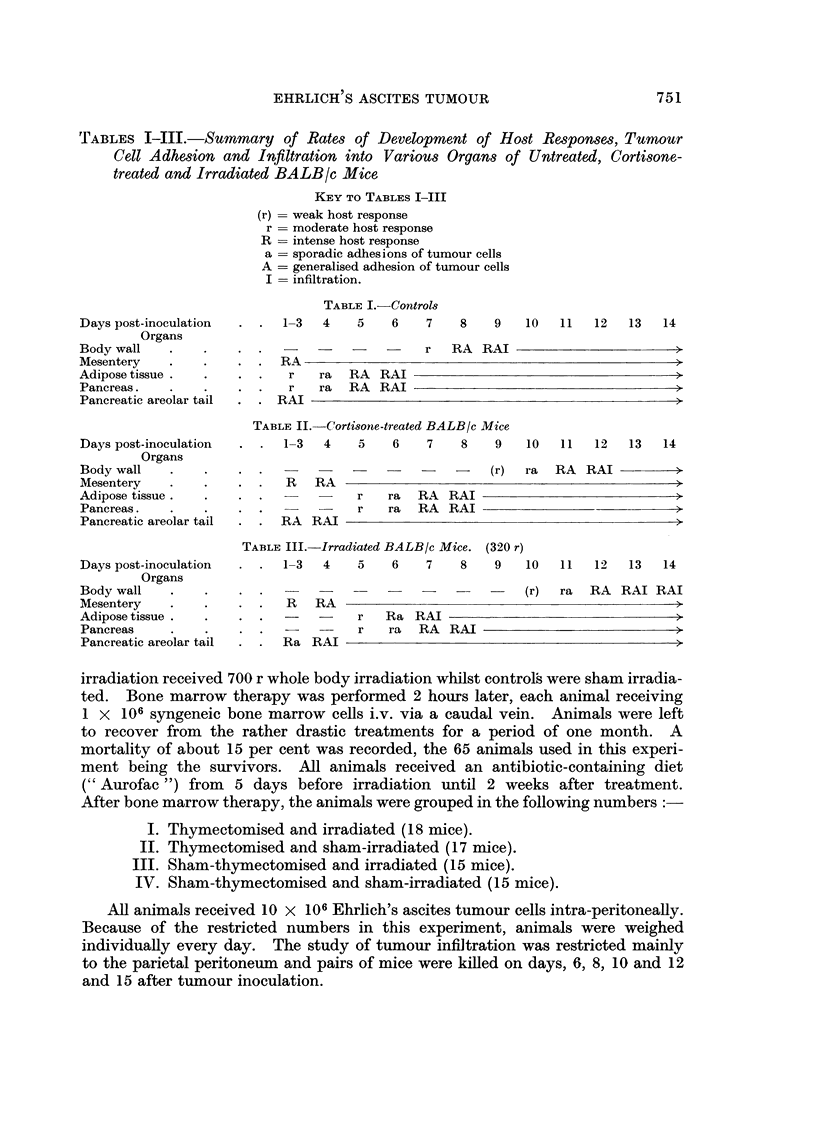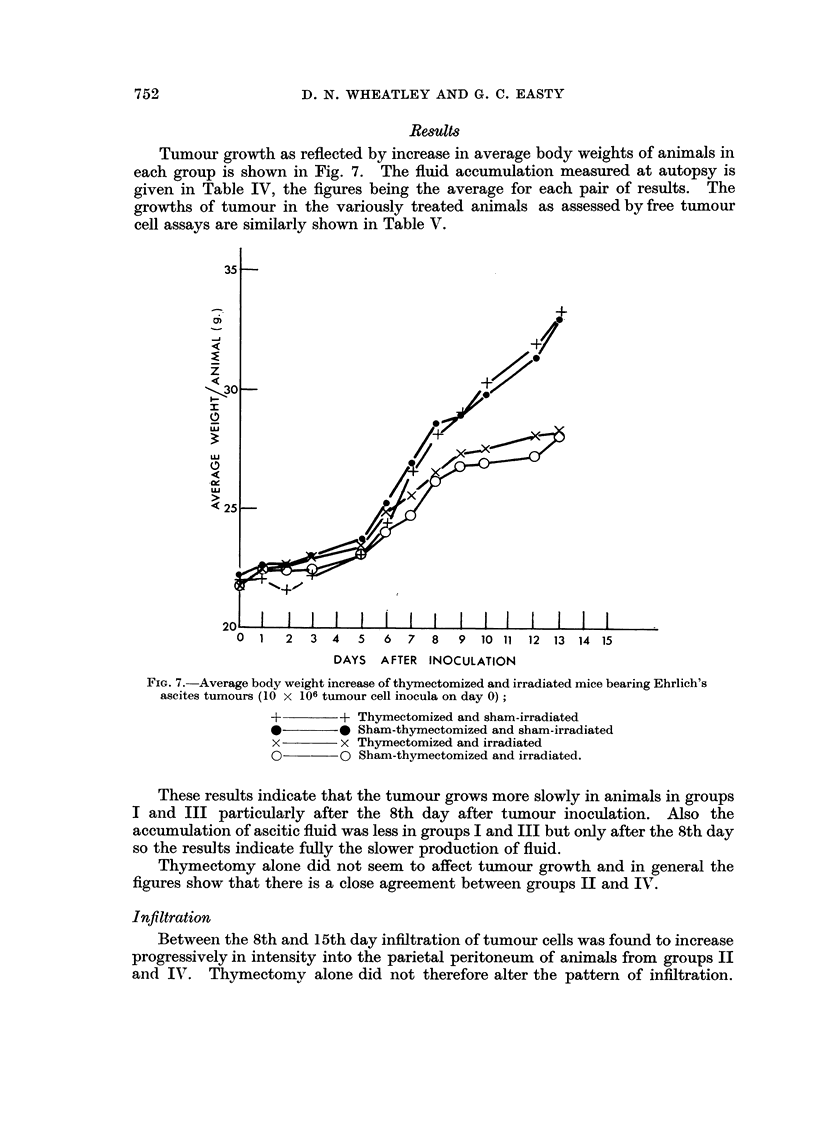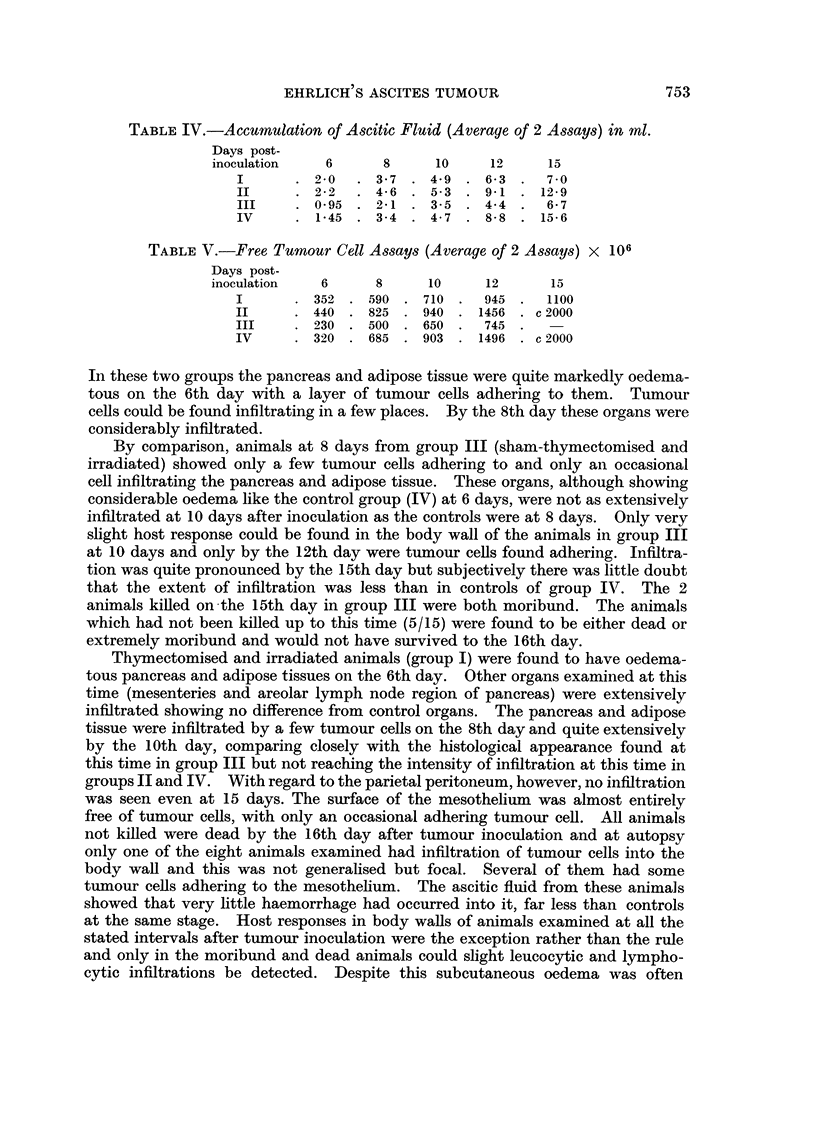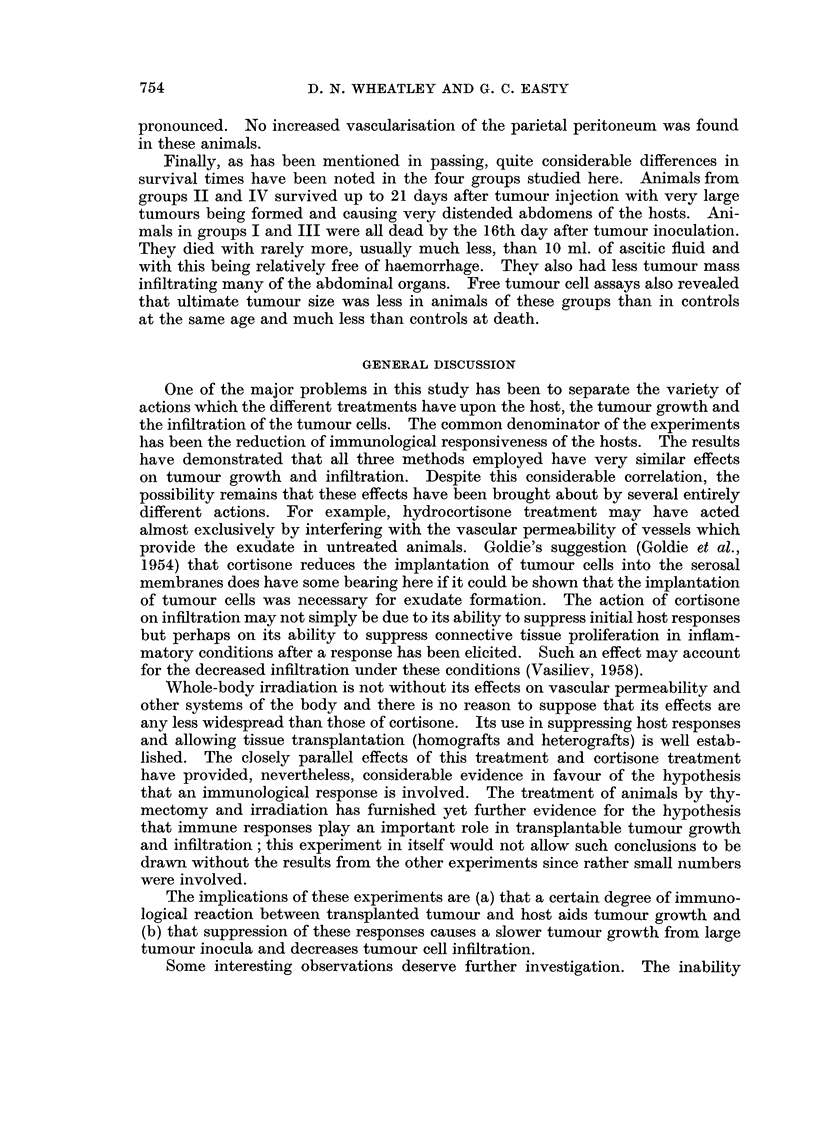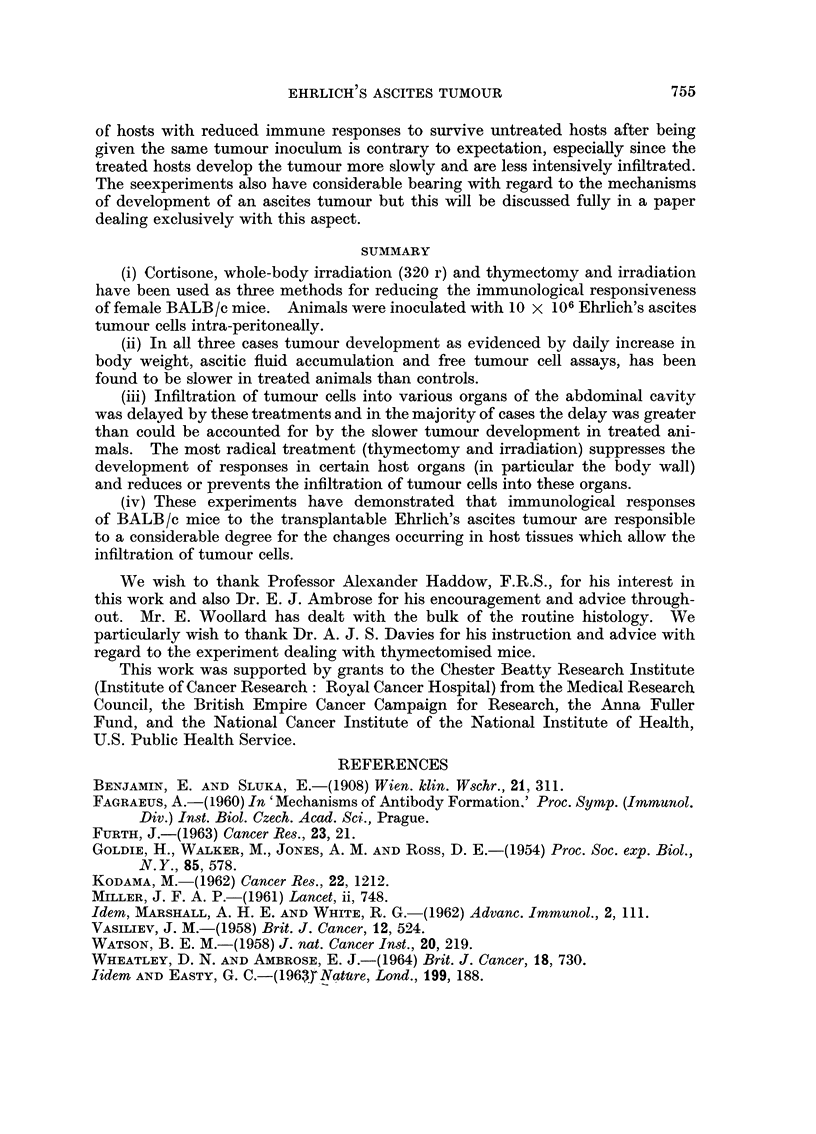# The Growth and Infiltration of Ehrlich's Ascites Tumour in Mice with Reduced Immunological Responses

**DOI:** 10.1038/bjc.1964.86

**Published:** 1964-12

**Authors:** D. N. Wheatley, G. C. Easty


### THE GROWTH AND INFILTRATION OF EHRLICH'S ASCITES TUMOUR IN MICE WITH REDUCED IMMUNOLOGICAL RESPONSES

D. N. WHEATLEY* AND G. C. EASTY

*From the Chester Beatty Research Institute, Institute of Cancer Research ; Royal Cancer Hospital, Fulham Road, London, S.W.3*

Received for publication May 4, 1964

RECENT reports have suggested that an immunological mechanism is involved in the tissue responses of mice to transplantable ascites tumours and that these responses give rise to conditions which support the infiltration of tumour cells (Wheatley, Ambrose and Easty, 1963 ; Wheatley and Ambrose, 1964). To investigate the role of immune responses in tumour cell invasion from ascites tumours, several different treatments known to reduce the immune responses of host animals have been carried out and the effects on tumour growth and infiltration have been studied. They are :

(i) Cortisone treatment,
(ii) Whole-body irradiation (sublethal), and
(iii) Thymectomy and irradiation.

### (i) THE EFFECT OF CORTISONE ON EHRLICH'S ASCITES TUMOUR GROWTH AND INFILTRATION

One way of encouraging tumour growth in a host which reacts to a transplanted homologous (or heterologous) tumour is to suppress the host response by treatment with cortisone. In the case of the Ehrlich's ascites tumour in BALB/c mice, low level inocula ($10^2$–$10^4$ tumour cells) have produced tumours rapidly in cortisone-treated animals whilst in untreated animals, these tumour cell inocula produce few ascites tumours.

On the other hand, several investigators have found that with a large tumour cell inoculum which gives a well defined outgrowth of ascites tumour in all hosts, cortisone treatment reduces the growth rate (Goldie *et al.*, 1954 ; Watson, 1958 ; Kodama, 1962). Goldie *et al.* (1954) have suggested that cortisone exerts this effect because it decreases both the serosal implantation of tumour cells (this being necessary for the production of exudate) and the vascular permeability thereby slowing down the rate of exudate formation.

### *Materials and Methods*

Eighty BALB/c mice (female) weighing between 20 and 25 grams and of 12–14 weeks were used. They were randomly sorted into 16 boxes of 5.

* Present address: Department of Pathology, University Medical Buildings, Foresterhill, Aberdeen.

(A) Twenty were given tumour and cortisone. Their body weights were followed through to death when all were autopsied.

(B) Thirty were given tumour and cortisone and were killed (usually 2 per day) for free tumour cell assay and histological study of infiltration.

(C) Twenty were given tumour and saline ; 12 of these were weighed daily and killed at regular intervals to check the infiltration and growth rate of the tumour alone.

(D) Ten were given cortisone but no tumour, and were weighed daily.

A further group E of 10 animals given saline alone showed no difference in body weight curve from group D over the period of the experiment and the graph has been omitted.

The steroid treatment was 3 mg. of hydrocortisone sodium succinate (Boots Pure Drug Co. Ltd.) per animal subcutaneously 3 days before tumour injection, and subsequently 1 mg./day/animal. The animals were pretreated so that their responses would be depressed before tumour was introduced (Fagraeus, 1960).

Ehrlich's ascites tumour of the near tetraploid was recovered from a BALB/c female host bearing an 8-day ascites tumour. The ascitic fluid was diluted with sterile saline (0·85 per cent) to give the appropriate tumour cell inoculum per animal in 0·2 ml. The inoculum was 10 million viable cells (checked by haemocytometer counts with viability tests performed concurrently by lissamine green exclusion as described previously (Wheatley and Ambrose, 1964)).

Tumour growth was measured overtly by weighing animals daily. Animals were killed 2 each day from the cortisone-treated group and 1 each day from the saline-treated tumour group. Tumour growth by free tumour cell assay was measured for these animals and histological sections were taken of the pancreas, pancreato-splenic lymph node region, mesenteries, adipose tissue, body wall, spleen and liver. Details of the methods have been previously described (Wheatley and Ambrose, 1964).

### *Results*

The body weight curves are shown in Fig. 1. It can be seen quite clearly that there was a slower body weight increase in cortisone-treated animals than in the saline-treated controls. Autopsies have revealed that there was far less accumulation of ascitic fluid in cortisone-treated animals. This is shown in Fig. 2.

Although the accumulation of ascitic fluid is slower in treated animals the proliferation of tumour cells is not *proportionately reduced.* Thus in the cortisone-treated animals the smaller amounts of ascitic fluid usually contain far greater concentrations of tumour cells. The difference between the two groups with regard to tumour assay is shown in Fig. 3.

### *Infiltration*

The control tumour group showed a pattern of infiltration in accordance with earlier results (Wheatley and Ambrose, 1964), the mesenteries and pancreatic lymph node complex being invaded most rapidly, then the pancreas and adipose tissue and, by the 9th day of tumour development, the parietal peritoneum. Infiltration in the treated animals can be compared with control results on two separate grounds: (a) in relation to time after tumour inoculation and (b) at comparable tumour assays. With regard to time after inoculation, it was found that infiltration was delayed by 2 or 3 days in the treated animals, except in the mesenteries and the areolar region of the pancreato-splenic lymph node complex where the delay was of the order of 1 day. The body wall was infiltrated from the 12th day post-inoculum in cortisone-treated hosts whilst in controls infiltration was apparent from the 9th day onwards. Cortisone reduced the vascularisa-

FIG. 1.—Increase in body weights of groups of BALB/c mice given 10 × $10^6$ Ehrlich's ascites tumour cells and hydrocortisone (1 mg./day). Controls were given saline only.

○ Ehrlich and saline.
× Ehrlich and cortisone.
● Cortisone only.

tion of the peritoneal surfaces and reduced the haemorrhagic conditions in the later stages of tumour development. For summary see Tables I and II.

In comparing cortisone-treated and untreated animals bearing approximately equal free tumour cell numbers, the delay in the onset of tumour cell infiltration was found to be less pronounced than in the first comparison since tumour cell proliferation was slower in the cortisone group. The infiltration into the mesenteries and areolar tissue occurred at about the same tumour size in cortisonetreated animals and saline-treated controls while the onset of infiltration into the pancreas or body wall did not occur until a larger tumour was present in treated animals.

It has been found, therefore, that cortisone depresses the host response to the tumour, reduces peritoneal exudate considerably and slows down the proliferation of tumour cells. Infiltration was delayed appreciably with regard to time after

FIG. 2.—Ascitic fluid production in animals similarly treated, the volumes being measured at autopsy.

O——O Untreated.
X——X Cortisone treated.

tumour inoculation but less so with regard to actual tumour size. The interpretation of these results is difficult since cortisone has so many widespread effects on the body (Furth, 1963). They will be discussed in relation to other work later in this paper.

### (ii) THE EFFECT OF WHOLE-BODY IRRADIATION OF HOSTS ON TUMOUR GROWTH AND INFILTRATION

Whole-body irradiation with X-rays has been known to reduce the capacity of an animal to produce antibody to a foreign protein since the experiments of Benjamin and Sluka in 1908. It was considered of interest to compare the effect of this treatment on Ehrlich's ascites tumour growth and infiltration with the effect which has been obtained with hydrocortisone.

*Materials and Methods*

Seventy mice were used in this experiment, of the same strain, sex and age as for the cortisone experiment. Animals were fed an antibiotic—containing commercial diet ("Aurofac") from 4 days before irradiation to one week after irradiation. Irradiation was carried out using a Marconi 250 kv 15 mA therapeutic X-ray machine with a $\frac{1}{2}$ mm. Cu + 1 mm. Al filter. The exposure rate

FIG. 3.—Free tumour cell assays for animals also similarly treated, measured at autopsy.
× Cortisone treated.
● Saline control.

was 29 r/min. and the animals received 320 r. All sham-irradiated animals received the same treatment but without the machine in operation.

Tumour cells ($10^7$ cells per animal) were injected within 2 hours of irradiation. Animals were weighed every day from 2 days before irradiation. The various groups were as follows :

(a) Irradiated + tumour, 30 animals, divided 10 for body weight studies to death and 20 killed at daily intervals from 3 days, usually 2 per day.

(b) Irradiated only, 10 animals.

(c) Sham-irradiated + tumour, 20 animals, 10 for body weight studies to death and 10 killed at regular intervals to check tumour growth and infiltration as controls.

(d) Sham-irradiated only, 10 animals.

Tumour assays were performed on all animals killed and the organs removed for histological study were as for the cortisone experiment.

*Results*

The growth of ascites tumour in irradiated host has been found to be slower than in controls, as shown by the body weight curves in Fig. 4 and also by the difference in ascitic fluid production in Fig. 5. Free tumour cell assays, however, show that there is less difference in actual tumour cell proliferation rates between irradiated hosts and sham-irradiated control (Fig. 6). The decreased rate of tumour cell proliferation compares closely with the rate of proliferation in cortisone-treated animals. Tumours in the irradiated hosts were not as haemorrhagic as in controls and at late stages the peritoneal surfaces were less vascularised.

FIG. 4.—Increase in body weights of groups of BALB/c mice given $10 \times 10^6$ Ehrlich's ascites tumour cells on Day 0 (↑) following whole-body x-irradiation (320 r) or sham-irradiation.

×———× Irradiated + tumour.

×– – – –× Irradiated + tumour.

●———● Sham irradiated + tumour.

○———○ Irradiated + saline.

*Infiltration*

Host responses, tumour cell adhesion and infiltration in irradiated animals is delayed and minimised as compared with controls. The times of onset of the various processes in the host tissues selected for study are shown in Table III on page 751. Again, the delay in response and tumour cell adhesion to the mesenteries was not found to be greater than 1 day, but in the pancreas and adipose tissue response to the ascitic tumour, tumour cell adhesion and infiltration were delayed by between 2 and 3 days. The body wall does not support tumour cell adhesion until 12 days after tumour inoculation whilst the controls are infiltrated at 9 days. In terms of equivalent tumour growths, as with cortisone treatment,

Fig. 5.—Ascitic fluid production in animals similarly treated, the volumes being measured at autopsy.

O——————O Sham-irradiated.
X– – – – – –X Irradiated.

it appears that irradiation delays the onset of infiltration into host tissues. The delay in infiltration of the pancreas, adipose tissue and parietal peritoneum is greater, therefore, than would be expected from the retardation of tumour growth alone in irradiated animals compared with the controls. For summary see Table III.

Briefly, whole body irradiation of mice as performed in the experiment described here, has a remarkably similar effect on Ehrlich's ascites tumour growth to that obtained by cortisone treatment. It is therefore considered more plausible that immunological reactions are involved in tumour cell infiltration from ascites tumours since two quite separate treatments known to depress such responses have exerted such similar effects. A fuller discussion of these results is to be found later.

### (iii) THE EFFECT OF THYMECTOMY AND IRRADIATION OF HOSTS ON EHRLICH'S ASCITES TUMOUR GROWTH AND INFILTRATION

The realisation of the possible role of the thymus in immunological responsiveness (Miller, 1961 ; Miller, Marshall and White, 1962) has led to the development of the technique of thymectomy and irradiation to abrogate the immunological

FIG. 6.—Free tumour cell assays for animals also similarly treated, as measured at autopsy.
O——O Control.
×——× Irradiated.

responsiveness of animals. The technique involves treatment of animals with lethal doses of X-rays and to restore the vitality of the subjects, syngeneic bone marrow cells are injected. Thus the effect of immunological unresponsiveness on ascites tumour growth and infiltration can be investigated more critically than by the previous methods.

*Materials and Methods*

Sixty-five BALB/c female mice were used, and for this study they were 3 months of age before treatment was carried out. All weighed approximately 25 g.

Thymectomy was carried out under ether anaesthesia. Control animals were sham thymectomised, undergoing the complete surgery except for the removal of the thymus itself. One week following thymectomy, animals designated for

TABLES I–III.—*Summary of Rates of Development of Host Responses, Tumour Cell Adhesion and Infiltration into Various Organs of Untreated, Cortisone-treated and Irradiated BALB/c Mice*

KEY TO TABLES I–III

(r) = weak host response
r = moderate host response
R = intense host response
a = sporadic adhesions of tumour cells
A = generalised adhesion of tumour cells
I = infiltration.

TABLE I.—*Controls*

| Days post-inoculation | 1–3 | 4 | 5 | 6 | 7 | 8 | 9 | 10 | 11 | 12 | 13 | 14 |
|---|---|---|---|---|---|---|---|---|---|---|---|---|
| Organs | | | | | | | | | | | | |
| Body wall | — | — | — | — | r | RA | RAI | → | | | | |
| Mesentery | RA | → | | | | | | | | | | |
| Adipose tissue | r | ra | RA | RAI | → | | | | | | | |
| Pancreas | r | ra | RA | RAI | → | | | | | | | |
| Pancreatic areolar tail | RAI | → | | | | | | | | | | |

TABLE II.—*Cortisone-treated BALB/c Mice*

| Days post-inoculation | 1–3 | 4 | 5 | 6 | 7 | 8 | 9 | 10 | 11 | 12 | 13 | 14 |
|---|---|---|---|---|---|---|---|---|---|---|---|---|
| Organs | | | | | | | | | | | | |
| Body wall | — | — | — | — | — | — | (r) | ra | RA | RAI | → | |
| Mesentery | R | RA | → | | | | | | | | | |
| Adipose tissue | — | — | r | ra | RA | RAI | → | | | | | |
| Pancreas | — | — | r | ra | RA | RAI | → | | | | | |
| Pancreatic areolar tail | RA | RAI | → | | | | | | | | | |

TABLE III.—*Irradiated BALB/c Mice.* (320 *r*)

| Days post-inoculation | 1–3 | 4 | 5 | 6 | 7 | 8 | 9 | 10 | 11 | 12 | 13 | 14 |
|---|---|---|---|---|---|---|---|---|---|---|---|---|
| Organs | | | | | | | | | | | | |
| Body wall | — | — | — | — | — | — | — | (r) | ra | RA | RAI | RAI |
| Mesentery | R | RA | → | | | | | | | | | |
| Adipose tissue | — | — | r | Ra | RAI | → | | | | | | |
| Pancreas | — | — | r | ra | RA | RAI | → | | | | | |
| Pancreatic areolar tail | Ra | RAI | → | | | | | | | | | |

irradiation received 700 r whole body irradiation whilst controls were sham irradiated. Bone marrow therapy was performed 2 hours later, each animal receiving $1 \times 10^6$ syngeneic bone marrow cells i.v. via a caudal vein. Animals were left to recover from the rather drastic treatments for a period of one month. A mortality of about 15 per cent was recorded, the 65 animals used in this experiment being the survivors. All animals received an antibiotic-containing diet (" Aurofac ") from 5 days before irradiation until 2 weeks after treatment. After bone marrow therapy, the animals were grouped in the following numbers :—

I. Thymectomised and irradiated (18 mice).
II. Thymectomised and sham-irradiated (17 mice).
III. Sham-thymectomised and irradiated (15 mice).
IV. Sham-thymectomised and sham-irradiated (15 mice).

All animals received $10 \times 10^6$ Ehrlich's ascites tumour cells intra-peritoneally. Because of the restricted numbers in this experiment, animals were weighed individually every day. The study of tumour infiltration was restricted mainly to the parietal peritoneum and pairs of mice were killed on days, 6, 8, 10 and 12 and 15 after tumour inoculation.

### *Results*

Tumour growth as reflected by increase in average body weights of animals in each group is shown in Fig. 7. The fluid accumulation measured at autopsy is given in Table IV, the figures being the average for each pair of results. The growths of tumour in the variously treated animals as assessed by free tumour cell assays are similarly shown in Table V.

FIG. 7.—Average body weight increase of thymectomized and irradiated mice bearing Ehrlich's ascites tumours (10 × $10^6$ tumour cell inocula on day 0) ;

+——+ Thymectomized and sham-irradiated
●——● Sham-thymectomized and sham-irradiated
×——× Thymectomized and irradiated
○——○ Sham-thymectomized and irradiated.

These results indicate that the tumour grows more slowly in animals in groups I and III particularly after the 8th day after tumour inoculation. Also the accumulation of ascitic fluid was less in groups I and III but only after the 8th day so the results indicate fully the slower production of fluid.

Thymectomy alone did not seem to affect tumour growth and in general the figures show that there is a close agreement between groups II and IV.

### *Infiltration*

Between the 8th and 15th day infiltration of tumour cells was found to increase progressively in intensity into the parietal peritoneum of animals from groups II and IV. Thymectomy alone did not therefore alter the pattern of infiltration.

TABLE IV.—*Accumulation of Ascitic Fluid (Average of 2 Assays) in ml.*

| Days post-inoculation | 6 | 8 | 10 | 12 | 15 |
|---|---|---|---|---|---|
| I | 2·0 | 3·7 | 4·9 | 6·3 | 7·0 |
| II | 2·2 | 4·6 | 5·3 | 9·1 | 12·9 |
| III | 0·95 | 2·1 | 3·5 | 4·4 | 6·7 |
| IV | 1·45 | 3·4 | 4·7 | 8·8 | 15·6 |

TABLE V.—*Free Tumour Cell Assays (Average of 2 Assays)* $\times 10^6$

| Days post-inoculation | 6 | 8 | 10 | 12 | 15 |
|---|---|---|---|---|---|
| I | 352 | 590 | 710 | 945 | 1100 |
| II | 440 | 825 | 940 | 1456 | *c* 2000 |
| III | 230 | 500 | 650 | 745 | — |
| IV | 320 | 685 | 903 | 1496 | *c* 2000 |

In these two groups the pancreas and adipose tissue were quite markedly oedematous on the 6th day with a layer of tumour cells adhering to them. Tumour cells could be found infiltrating in a few places. By the 8th day these organs were considerably infiltrated.

By comparison, animals at 8 days from group III (sham-thymectomised and irradiated) showed only a few tumour cells adhering to and only an occasional cell infiltrating the pancreas and adipose tissue. These organs, although showing considerable oedema like the control group (IV) at 6 days, were not as extensively infiltrated at 10 days after inoculation as the controls were at 8 days. Only very slight host response could be found in the body wall of the animals in group III at 10 days and only by the 12th day were tumour cells found adhering. Infiltration was quite pronounced by the 15th day but subjectively there was little doubt that the extent of infiltration was less than in controls of group IV. The 2 animals killed on the 15th day in group III were both moribund. The animals which had not been killed up to this time (5/15) were found to be either dead or extremely moribund and would not have survived to the 16th day.

Thymectomised and irradiated animals (group I) were found to have oedematous pancreas and adipose tissues on the 6th day. Other organs examined at this time (mesenteries and areolar lymph node region of pancreas) were extensively infiltrated showing no difference from control organs. The pancreas and adipose tissue were infiltrated by a few tumour cells on the 8th day and quite extensively by the 10th day, comparing closely with the histological appearance found at this time in group III but not reaching the intensity of infiltration at this time in groups II and IV. With regard to the parietal peritoneum, however, no infiltration was seen even at 15 days. The surface of the mesothelium was almost entirely free of tumour cells, with only an occasional adhering tumour cell. All animals not killed were dead by the 16th day after tumour inoculation and at autopsy only one of the eight animals examined had infiltration of tumour cells into the body wall and this was not generalised but focal. Several of them had some tumour cells adhering to the mesothelium. The ascitic fluid from these animals showed that very little haemorrhage had occurred into it, far less than controls at the same stage. Host responses in body walls of animals examined at all the stated intervals after tumour inoculation were the exception rather than the rule and only in the moribund and dead animals could slight leucocytic and lymphocytic infiltrations be detected. Despite this subcutaneous oedema was often pronounced. No increased vascularisation of the parietal peritoneum was found in these animals.

Finally, as has been mentioned in passing, quite considerable differences in survival times have been noted in the four groups studied here. Animals from groups II and IV survived up to 21 days after tumour injection with very large tumours being formed and causing very distended abdomens of the hosts. Animals in groups I and III were all dead by the 16th day after tumour inoculation. They died with rarely more, usually much less, than 10 ml. of ascitic fluid and with this being relatively free of haemorrhage. They also had less tumour mass infiltrating many of the abdominal organs. Free tumour cell assays also revealed that ultimate tumour size was less in animals of these groups than in controls at the same age and much less than controls at death.

### GENERAL DISCUSSION

One of the major problems in this study has been to separate the variety of actions which the different treatments have upon the host, the tumour growth and the infiltration of the tumour cells. The common denominator of the experiments has been the reduction of immunological responsiveness of the hosts. The results have demonstrated that all three methods employed have very similar effects on tumour growth and infiltration. Despite this considerable correlation, the possibility remains that these effects have been brought about by several entirely different actions. For example, hydrocortisone treatment may have acted almost exclusively by interfering with the vascular permeability of vessels which provide the exudate in untreated animals. Goldie's suggestion (Goldie *et al.*, 1954) that cortisone reduces the implantation of tumour cells into the serosal membranes does have some bearing here if it could be shown that the implantation of tumour cells was necessary for exudate formation. The action of cortisone on infiltration may not simply be due to its ability to suppress initial host responses but perhaps on its ability to suppress connective tissue proliferation in inflammatory conditions after a response has been elicited. Such an effect may account for the decreased infiltration under these conditions (Vasiliev, 1958).

Whole-body irradiation is not without its effects on vascular permeability and other systems of the body and there is no reason to suppose that its effects are any less widespread than those of cortisone. Its use in suppressing host responses and allowing tissue transplantation (homografts and heterografts) is well established. The closely parallel effects of this treatment and cortisone treatment have provided, nevertheless, considerable evidence in favour of the hypothesis that an immunological response is involved. The treatment of animals by thymectomy and irradiation has furnished yet further evidence for the hypothesis that immune responses play an important role in transplantable tumour growth and infiltration ; this experiment in itself would not allow such conclusions to be drawn without the results from the other experiments since rather small numbers were involved.

The implications of these experiments are (a) that a certain degree of immunological reaction between transplanted tumour and host aids tumour growth and (b) that suppression of these responses causes a slower tumour growth from large tumour inocula and decreases tumour cell infiltration.

Some interesting observations deserve further investigation. The inability of hosts with reduced immune responses to survive untreated hosts after being given the same tumour inoculum is contrary to expectation, especially since the treated hosts develop the tumour more slowly and are less intensively infiltrated. The seexperiments also have considerable bearing with regard to the mechanisms of development of an ascites tumour but this will be discussed fully in a paper dealing exclusively with this aspect.

### SUMMARY

(i) Cortisone, whole-body irradiation (320 r) and thymectomy and irradiation have been used as three methods for reducing the immunological responsiveness of female BALB/c mice. Animals were inoculated with $10 \times 10^6$ Ehrlich's ascites tumour cells intra-peritoneally.

(ii) In all three cases tumour development as evidenced by daily increase in body weight, ascitic fluid accumulation and free tumour cell assays, has been found to be slower in treated animals than controls.

(iii) Infiltration of tumour cells into various organs of the abdominal cavity was delayed by these treatments and in the majority of cases the delay was greater than could be accounted for by the slower tumour development in treated animals. The most radical treatment (thymectomy and irradiation) suppresses the development of responses in certain host organs (in particular the body wall) and reduces or prevents the infiltration of tumour cells into these organs.

(iv) These experiments have demonstrated that immunological responses of BALB/c mice to the transplantable Ehrlich's ascites tumour are responsible to a considerable degree for the changes occurring in host tissues which allow the infiltration of tumour cells.

We wish to thank Professor Alexander Haddow, F.R.S., for his interest in this work and also Dr. E. J. Ambrose for his encouragement and advice throughout. Mr. E. Woollard has dealt with the bulk of the routine histology. We particularly wish to thank Dr. A. J. S. Davies for his instruction and advice with regard to the experiment dealing with thymectomised mice.

This work was supported by grants to the Chester Beatty Research Institute (Institute of Cancer Research : Royal Cancer Hospital) from the Medical Research Council, the British Empire Cancer Campaign for Research, the Anna Fuller Fund, and the National Cancer Institute of the National Institute of Health, U.S. Public Health Service.

### REFERENCES

BENJAMIN, E. AND SLUKA, E.—(1908) *Wien. klin. Wschr.*, **21**, 311.

FAGRAEUS, A.—(1960) *In* 'Mechanisms of Antibody Formation.' *Proc. Symp.* (*Immunol. Div.*) *Inst. Biol. Czech. Acad. Sci.*, Prague.

FURTH, J.—(1963) *Cancer Res.*, **23**, 21.

GOLDIE, H., WALKER, M., JONES, A. M. AND ROSS, D. E.—(1954) *Proc. Soc. exp. Biol., N.Y.*, **85**, 578.

KODAMA, M.—(1962) *Cancer Res.*, **22**, 1212.

MILLER, J. F. A. P.—(1961) *Lancet*, ii, 748.

*Idem*, MARSHALL, A. H. E. AND WHITE, R. G.—(1962) *Advanc. Immunol.*, **2**, 111.

VASILIEV, J. M.—(1958) *Brit. J. Cancer*, **12**, 524.

WATSON, B. E. M.—(1958) *J. nat. Cancer Inst.*, **20**, 219.

WHEATLEY, D. N. AND AMBROSE, E. J.—(1964) *Brit. J. Cancer*, **18**, 730.

*Iidem* AND EASTY, G. C.—(1963) *Nature, Lond.*, **199**, 188.